# New medical treatments for lower urinary tract symptoms due to benign prostatic hyperplasia and future perspectives

**DOI:** 10.1186/s12894-016-0176-0

**Published:** 2016-09-15

**Authors:** Simone Albisinni, Ibrahim Biaou, Quentin Marcelis, Fouad Aoun, Cosimo De Nunzio, Thierry Roumeguère

**Affiliations:** 1 Urology Department, Erasme Hospital, Université Libre de Bruxelles, Route de Lennik 808, B-1070 Brussels, Belgium; 2 Department of Urology, Ospedale Sant’Andrea, University “La Sapienza”, Roma, Italy

**Keywords:** Benign prostatic hyperplasia, Medical treatment, Prostate

## Abstract

**Background:**

Lower Urinary Tract Symptoms (LUTS) in men are a common clinical problem in urology and have been historically strictly linked to benign prostatic hyperplasia (BPH), which may lead to bladder outlet obstruction (BOO). New molecules have been approved and have entered the urologists’ armamentarium, targeting new signaling pathways and tackling specific aspects of LUTS. Objective of this review is to summarize the evidence regarding the new medical therapies currently available for male non-neurogenic LUTS, including superselective α1-antagonists, PDE-5 inhibitors, anticholinergic drugs and intraprostatic onabotulinum toxin injections.

**Methods:**

The National Library of Medicine Database was searched for relevant articles published between January 2006 and December 2015, including the combination of “BPH”, “LUTS”, “medical” and “new”. Each article’s title, abstract and text were reviewed for their appropriateness and their relevance. One hundred forty eight articles were reviewed.

**Results:**

Of the 148 articles reviewed, 92 were excluded. Silodosin may be considered a valid alternative to non-selective α1-antagonists, especially in the older patients where blood pressure alterations may determine major clinical problems and ejaculatory alterations may be not truly bothersome. Tadalafil 5 mg causes a significant decrease of IPSS score with an amelioration of patients’ QoL, although with no significant increase in Q_max_. Antimuscarinic drugs are effective on storage symptoms but should be used with caution in patients with elevated post-void residual. Intraprostatic injections of botulinum toxin are well-tolerated and effective, with a low rate of adverse events; however profound ameliorations were seen also in the sham arms of RCTs evaluating intraprostatic injections.

**Conclusion:**

New drugs have been approved in the last years in the medical treatment of BPH-related LUTS. Practicing urologists should be familair with their pharmacodynamics and pharmacokinetics.

## Background

Lower Urinary Tract Symptoms (LUTS) in men are a common clinical problem in urology, and have been historically strictly linked to benign prostatic hyperplasia (BPH). These are classified into storage, voiding and post micturition symptoms [1]. However, BPH does not describe symptoms, but is instead a histologic diagnosis, characterized by a micronodular hyperplasia evolving into a macroscopic nodular enlargement, which in turn may determine bladder outlet obstruction (BOO). Although BOO as a consequence of BPH may be responsible for a part of male LUTS, studies have found that the prostate is not the only actor in the complex play of male LUTS. The bladder and it’s articulated neuronal control has been found to be another main character in this plot [2]. To support this theory, also women suffer from storage LUTS, with overactive bladder (OAB) being the most frequent cause. Moreover, although voiding LUTS are the most common symptoms in BPH, storage are the most bothersome with great impact on the patients’ quality of life (QoL) [3]. As such, today it is insufficient and inappropriate to consider the prostate as the only therapeutic target in the management of LUTS in men, even when BOO is present. Rather, the entire lower urinary tract, from the afferent sensory nerves to the urethra, must be seen as a whole and in this direction research is moving [4].

Historically, the standard medical treatment for LUTS in men with BPH included α1-antagonists, 5α-reductase inhibitors and phytotherapy. These agents remain indeed today the mainstay of BPH treatment. Nonetheless, albeit full dose treatment, some patients remain symptomatic or may experience BPH progression, defined as the onset of acute urinary retention (AUR), urinary infection (UI) or the need of BPH-related surgery [5]. In addition, the drugs routinely used in the management of LUTS carry potential adverse effects (AE), which in turn may be the cause of non-compliance of patients [6]. Therefore, research is progressing in order to expand and optimize medical strategies in the management of BPH-related LUTS. Selective α1-antagonists, phosphodiesterase 5 (PDE5) inhibitors, and anticholinergics have been tested and have entered our armamentarium for the management of male LUTS. These agents, their pharmacodynamics, pharmacokinetics and AEs should be well known to the practicing urologist. Furthermore, our knowledge of bladder and prostatic molecular anatomy is constantly growing, and in parallel new biomolecular targets are being identified and explored as new candidates in BPH management. Objective of this systematic review is to summarize the evidence regarding the new medical therapies currently available for BPH-related LUTS, and to give an overview on current research and agents which may enter our everyday clinical practice in the close future.

## Methods

**Fig. 1:**
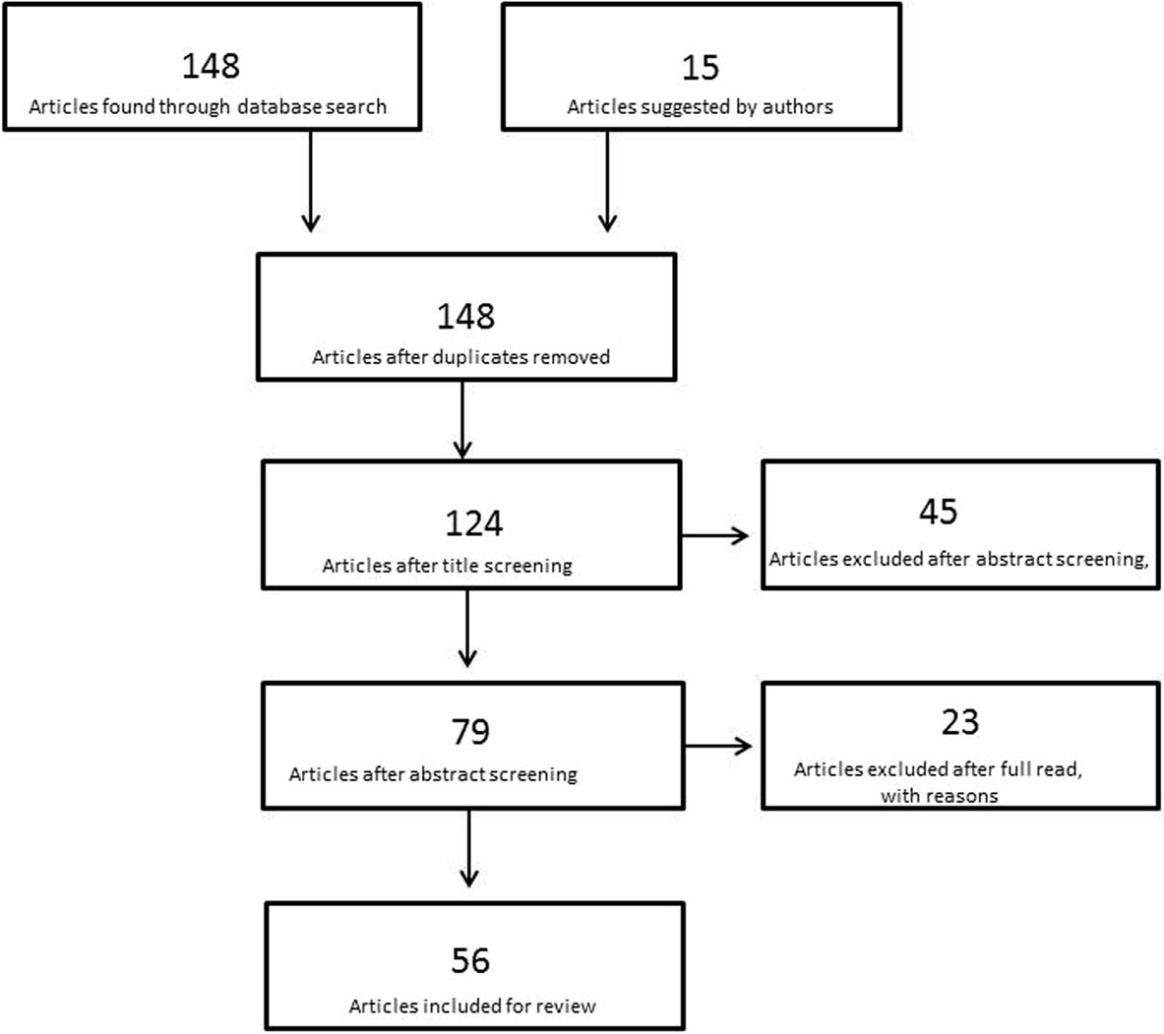
Flowchart of literature search

The National Library of Medicine Database was searched for relevant articles published between January 2006 and December 2015. A wide search was performed including the combination of following words: “BPH”, “LUTS”, “medical” “new”. Although recent articles were prioritized, manuscripts with relevant historical findings were referenced if necessary. Publications in English language were preferred, though if necessary data was extrapolated even from manuscripts in other languages. Evidence was not limited to human data; results from animal and in vitro experiments were also included in the review. Helsinki declaration principles were respected and informed consent was obtained. Each article’s title, abstract and text were reviewed for their appropriateness and their relevance. The initial list of selected papers was enriched by individual suggestions of the authors of the present review. Overall, 148 articles were reviewed. Of these, 92 were excluded after screening by the authors, leaving 56 articles eligible for the review (Fig. 1).

## Results and Discussion

### Selective α1-antagonists

α1-receptors are highly concentrated along the urinary and ejaculatory tracts [7], and non-selective α1-antagonists, alfuzosin, doxazosin, terazosin and tamsulosine are today the first line medical treatment in men with moderate to severe symptoms of BPH [8]. There are three main subtypes of α1-receptors expressed in the human organism: α1_A_, α1_B_ and α1_D_-receptors. These are all composed of seven transmembrane domains and are coupled with G proteins, and their stimulation results in the activation of phospholipase C with consequent increase in intracellular Ca^2+^, which in turn stimulates contraction in smooth muscular cells [7]. While α1_B_-receptors are typically found in vascular tissue, where they mediate arterial contraction, α1_A_ and α1_D_ are more specific of the lower urinary tract [7]. Kojima et al. explored the expression of these two subtypes in the transitional zone of 28 prostatic tissue of men affected by BPH, and found that 43 % were α1_A_ dominant, whereas 57 % α1_D_dominant [9]. These receptors are found also in the human detrusor muscle and in the spinal cord, although the role of these localizations in the pathology of LUTS remains controversial. Moreover, in a rat model, α1-receptors stimulation determined an increase in bladder vascular resistance, with doxazosin determining an increase in bladder blood flow [10].

Non-selective α1-antagonists act on the dynamic component of BPH, counteracting smooth muscle contraction in the prostate, which is augmented in BPH, with a consequent increase in urinary flow, reduction of LUTS and improvement in QoL [11, 12]. A recent metanalysis has demonstrated the reduction of bladder outlet obstruction index of −30.45 for silodosin, effect which was higher than all other available α1-antagonists [13]. However, due to their concurrent action on α1_B_-receptors, their use is associated with vascular AE, notably orthostatic hypotension, headaches and dizziness [8].

Silodosin is the most recently developed, highly selective antagonist of α1_A_-receptors. Its selectivity towards α1_A_-receptor blockade was reported to be 38 times higher than tamsulosin [13]. It has been shown that in vitro silodosin possesses an elevated α1_A_/α1_B_ binding ratio of 162/1 [14], and in vivo experiments have demonstrated its higher affinity for the urinary tract compared to the vascular system [15]. Kobayashi et al. demonstrated that in dogs, while tamsulosin inhibits intraurethral pressure in a dose dependent manner with a concurrent reduction of blood pressure (especially in old dogs), silodosin determines similar effects on intraurethral pressures without altering blood pressure [16]. The recommended dosage is 8 mg once –daily, which has been found to be non-inferior to 4 mg twice-daily in a double-blind randomized controlled trial (RCT) [17]. After administration, Silodosin is quickly absorbed and has a bioavailability of 32 % at 8 mg/day (therapeutic dose) [18]. Tmax is reached in 2.6 h and half life of the drug is 13.3 h. The drug is then eliminated via fecal (55 %) and renal (45 %) route [18].

On this pharmacologic basis, silodosin has been tested in order to evaluate its non-inferior effect on BPH, while minimizing peripheral vasodilatation and cardiovascular effects which may be cause of falls and fractures, especially in the elderly [19]. Chapple et al. have explored the efficacy of silodosin in a prospective, placebo controlled trial [20]. Nine hundred ninety five European men were randomized to receive either 8 mg silodosin, 0,4 mg tamsulosin or placebo on a daily basis for 12 weeks. The authors found a significant improvement of voiding and storage LUTS after treatment with silodosin compared to placebo (Δ IPSS: 2.3, 95%CI 1.4-3.2, *p* < 0.001)*, similar to that of tamsulosin, with a significant amelioration of patients’ QoL. Furthermore, silodosin determined a reduction of nycturia (Change from baseline silodosin vs placebo: −0.9 vs-0.7, *p* = 0.013), effect which was non-significant (*p* = 0.095) for tamsulosin vs placebo. Silodosin also caused an increase in urinary flow of3.77 ml/s, though this was not significantly higher compared to placebo (*p* = 0.089). Kawabe et al. reported results of a RCT which included 457 Japanese men treated by silodosin, tamsulosin or placebo, and found a significant decrease in total IPSS in the silodosin arm compared to placebo [21]. Similarly, Marks et al. found that 8 mg daily silodosin caused a significant reduction of both storage and voiding LUTS compared to placebo (Δ total 1.9, *p* < 0.0001; Δ storage 0.5, *p* = 0.0002; Δ voiding 1.4 *p* < 0.0001) [22]. Finally, it appears that Silodosin may decrease nycturia, especially in patients >65 years old in which desmopressin therapy may be problematic: in a pooled analysis of three RCTs, Eisenhardt et al. found that silodosin, compared to placebo, determined a significant nicturia improvement (53.4 vs. 42.8 %, *p* < 0.0001) [23], making it an interesting drug in the elderly population.

Most studies reported that truly silodosin determines less peripheral vascular AE compared to non-selective α1-antagonists, as it does not determine significant effects on supine or orthotopic blood pressure [20, 24]. However, silodosin caused in most trials a higher rate of anejaculation compared to non-selective α1-antagonists or tamsulosin, with rates between 14.2 and 20.9 %: this has been hypothesized to be a consequence of its selectivity for α1_A_-receptors, which are highly expressed along the vas deferens, with a consequent reduction of their contractility [20]. In 30 young sexually active patients, Bozkurt et al. have found impaired ejaculation in 27/30 men, with significant enlargement of seminal vesicles [24]. A long-term analysis following 104 men for 6 years found a quite high discontinuation of silodosin (75 %), mostly due to progression of disease and need for surgery or to unknown causes; only 9/78 stopped treatment due to side effects [25].

As such, silodosin may be considered a valid alternative to non-selective α1-antagonists, especially in the older patients where blood pressure alterations may determine major clinical problems and ejaculatory alterations may be not truly bothersome.

### Phosphodiesterase 5 Inhibitors

In addition to adrenergic fibers, key actors in micturition are the nonadrenergic-noncholinergic fibers. This neural system is implicated in the release and increase of nitric oxide (NO), a cardinal molecule for intracellular signaling which causes an increase of cyclic guanosine monophosphate (cGMP), consequently catabolized by the enzyme PDE. After its fundamental discovery in the cavernous tissue and the development of PDE5 inhibitors in the treatment of erectile dysfunction (ED), researchers have demonstrated the presence of PDE-5 isoenzymes all along the lower urinary tract: they are in fact expressed in the detrusor, the prostate, the urethra and in pelvic vessels [26]. Here PDE-5 inhibition determines intracellular cGMP increase, which in turn may promote micturition via different though yet unclear mechanisms of action [27]. First, cGMP phosphorylates and inactivates a protein kinase G (ρ-kinase) to promote smooth muscle cells relaxation [28]. In addition, this ρ-kinase stimulates endothelin-1, which is a potent vasoconstrictor which can mediate muscle contraction [28]. Therefore, PDE-5 inhibition, via a cGMP increase, reduces ρ-kinase activity and thus increases relaxation in the lower urinary tract. Additionally, PDE inhibition enhances smooth muscle cell relaxation increasing NO activity as observed in the bladder neck, where nitrergic innervation is prominent [26], in the prostate, were PDE inhibition determines a dose-dependent tissue relaxation [29], and in urethral tissue [30]. Finally, PDE-5 inhibition leads to increased perfusion of pelvic organs with the hypothesis that pelvic atherosclerosis with consequent ischaemia may have a role in male LUTS [31], the vasodilatation and increased end-organ perfusion determined by PDE-5 inhibitors on blood vessels may be beneficial for LUTS. Of note, Bertolotto et al. demonstrated increased prostatic perfusion on contrast-enhanced ultrasound after administration of tadalafil 20 mg [32]. Moreover, soluble cGMP plays a key role in the NO-mediated inhibition of leukocyte rolling, and PDE5 inhibition may reduce atherosclerotic damage and overall inflammation by reducing leukocyte recruitment. Tadalafil was shown to attenuate in vitro the expression of the inflammatory cytokines TNF-α and IL-1βin pulmonary arteries [33] and of TNF-α and IL-8 in endothelial cells [34]. Finally, cGMP modulates afferent nerve fibers from the bladder and urethra, and PDE inhibitors may decrease the sensation of bladder filling, thus reducing urgency [26, 35]. In this context Minagawa et al. found that tadalafil significantly decreased afferent activity from the bladder in response to bladder filling in a rat model [35] and Behr-Roussel et al. reported a reduction of afferent signaling in rats with spinal cord injury in response to bladder filling after treatment with vardenafil [36].

Randomized, placebo-controlled clinical trials have demonstrated that daily treatment with 5 mg tadalafil improves safely BPH-related LUTS [37–39]. McVary et al. found that tadalafil determined a decrease of total IPSS of −3.8 compared to −1.7 with placebo after 12 weeks of treatment (*p* < 0.0001) [37]. QoL also significantly improved (−0.7 vs −0.3, *p* = 0.008), while no significant differences were observed for peak urinary flow (Q_max_). Roehrborn et al. confirmed these findings, and noted in their trial that the dose of tadalafil with the best risk-benefit profile was 5 mg, determining a reduction of IPSS of −4.87 vs −2.27 with placebo (*p* < 0.001) [38]. Similarly, also QoL was significantly improved with tadalafil 5 mg. On post hoc analysis, the authors found that although tadalafil caused a numerically superior increase in Q_max_ compared to placebo, this increase was non-significant [40]. Oelke et al. reported an amelioration of total IPSS after 12 weeks of tadalafil 5 mg (Δ IPSS vs placebo: −2.1, *p* = 0.001) and of QoL (−0.3, *p* = 0.022) [39]. Moreover, these investigators described a significant increase in Q_max_: +2.4 ml/s (tadalafil) vs +1.2 ml/s (placebo), *p* = 0.009: although this result is not consistent with those previously reported [41], it must be kept in mind that Q_max_ is intra individually variable and influenced by age, sexual activity and baseline Q_max_ severity [40]. In a meta-analysis, Gacci et al. synthesized that tadalafil determines a significant −2.85 decrease in overall IPSS compared to placebo and a significant −1.85 decrease in association with α1-inhibitors compared to α1-inhibitors alone [42]. Clearly, tadalafil also significantly improves erectile function with a net increase of the International Index of Erectile Function. Indeed, a point of controversy is whether the amelioration of IPSS and QoL, which are subjective measurements, is a direct consequence of tadalafil’s pharmacologic effect on the lower urinary tract or if the results observed are confounded by the fact that the patients, having an improved potency, are more sexually active and thus more satisfied. It appears however that the amelioration seen in IPSS and QoL is observed both in potent and impotent patients [43] and pooled data analyses determined that the LUTS amelioration was largely (92.5 %) determined by a direct effect of the drug [44]. Tadalafil is also being tested in combination therapy with tamsulosin [45] or finasteride [46], demonstrating a more pronounced amelioration of LUTS and ED symptoms in the combination arms. Concerning AE, the vast majority of manuscripts reported mild to moderate grade AE (dyspepsia and flushing), with a low rate (2–4 %) of discontinuation of the therapy secondary to AE [42, 47].

In conclusion, tadalafil 5 mg is an effective and well tolerated treatment for BPH-related LUTS, and is of cardinal importance when treating patients with concomitant ED. Tadalafil causes a significant decrease of IPSS score with an amelioration of patients’ QoL, although with no significant increase in Q_max_.

Great caution is advised when prescribing iPDE5. As a consequence of systemic vasodilation, reduced venous blood flow to the heart may trigger cardiac failure in patients with preexisting cardiac insufficiency [42]. As such, before prescribing iPDE5, the clinician must always exclude signs of cardiac insufficiency as dyspnea, lower extremity oedema, chest pain. Moreover, for the same pharmacologic reasons, concomitant treatment with nitroderivates is an absolute contraindication to iPDE5 utilisation [8].

### Antimuscarinics

Two main subtypes of muscarinic receptor (MR) are expressed in the lower urinary tract: M2 and M3 receptors. Their proportions in detrusor membranes are respectively evaluated at 71 % and 22 % [48]. While M3 are mainly responsible for detrusor contraction in both healthy and pathologic conditions, M2 are predominant in the urothelium and may be associated with pathologic changes in the bladder [49]. It has been commonly and reasonably thought that the main mechanism of action of antimuscarinics in the treatment of LUTS is mediated by a reduction of detrusor contractility. Specimens from patients with bladder overactivity are consistently denervated, and as such it has been hypothesized that possible denervation supersensibility to acetylcholine may be crucial in OAB physiopathology [50]. However MR are also present in the urothelium and are here involved in urothelial sensory function. The urothelium in fact releases multiple signal molecules, including acetylcholine, which activate unmyelinated afferent C-fibers present in the suburothelial layer of the bladder wall, and this release of acetylcholine is increased by bladder overstretching . This, associated to the denervation supersensitivity of the detrusor to acetylcholine, may induce disorganized contraction of small muscular units in the detrusor, generating pathologic afferent signals which in turn may determine urgency symptoms [51].

Antimuscarinics have been prevalently used in female patients with OAB: however today it is clear that in men with BPH, storage symptoms are partially caused by the bladder, with urodynamically proven OAB being a common cause [52]. As such antimuscarinic therapy has emerged as a new option in male LUTS management [8]. Chun-Hou Liao et al. have studied the predictors of therapeutic success with a first line antimuscarinic treatment in BPH men with predominant storage symptoms. In their 197 patients group, receiving tolterodine in monotherapy, higher baseline IPSS, higher baseline Qmax and lower prostate volume were each associated with a better response [53]. Treatment with antimuscarinics alone is still felt as dangerous in patients with BOO by many urologists, due to the possible increased risk of AUR. Abrams et al. reported that in men with mild to moderate BOO, the antimuscarinic tolterodine 2 mg twice daily for 12 weeks caused a significant increase in post void residual (PVR) urine compared to placebo (49 ml vs 16 ml): however, rates of AUR (3 %) and Q_max_ were equal across both groups [54]. This is probably a consequence of the action of antimuscarinics on the storage phase of micturition and not on voiding, as there is little evidence that these agents, at the therapeutic recommended doses, determine a significant reduction of voiding contraction [55]. Nonetheless, in daily clinical practice, the majority of patients is already under treatment with an α1-antagonist, and present with persisting storage symptoms. In this context, several trials have explored the efficacy and safety of the addition of an antimuscarinic to the α1-antagonist in these patients [56–58]. The TIMES study included 879 men with symptoms of BPH and OAB [56]. Patients were randomized to receive either tolterodine 4 mg ER + tamsuloin, one of the two drugs alone or placebo. After 12 weeks, in the combination arm the patients reported significant decrease in urgency incontinence episodes (−0.88 vs −0.31, *p* = 0.005), frequency (−2.54 vs −1.41, *p* < 0.001) and an amelioration of QoL. Although higher than for placebo and for tamsulosin alone, the rate of AUR was low for the combination (0.4 %) and the tolterodine arm alone (0.5 %). MacDiarmid et al. reported significant amelioration of both storage and voiding symptoms (*p* = 0.006) in men affected by BPH treated with tamsulosin + oxybutinin 10 mg, with a non-significant increase in PVR in treated patients compared to placebo [57]. Similarly, in the VICTOR study, 398 men were randomized to receive tamsulosin plus either solifenacin 5 mg or placebo. In the solifenacin group, patients showed a significant reduction of urgency episodes (−2.18 vs −1.10, *p* = 0.001) but a non-significant reduction of frequency (−1.05 vs −0.67, *p* = 0.135) [58]. In most trials the most frequent AE associated with antimuscarinics is xerostomia [56, 57]. Increase in PVR urine, though statistically significant in many studies, frequently did not determine a significant increase in the risk of AUR requiring catheterization [56, 57]. However, as recommended by current EAU guidelines, antimuscarinics are therefore medications which can be prescribed in men with BPH with residual storage symptoms after treatment with α1-antagonists. Before to start a treatment with an antimuscarinic, BPH patients should be monitored for PVR and then closely followed [8]. Some authors have been questioning the compliance to bi-therapy, considering the fact that the common chronic combination of antimuscarinics and αl-antagonists could be a burden for the patients. Barkin et al. reported a retrospective analysis based on patients prescriptions reimbursement data. They concluded that patients treated in combination therapy showed an improved persistence over a year period, compared to those on αl-antagonists monotherapy [59].

Concerning anticholinergic drugs, great care is necessary when prescribing these drugs in the elderly, as cognitive deterioration may be a serious consequence and one must bear in mind that 16 % of patients >70 years show some form of cognitive impairment [60]. Indeed, encephalic cholinergic activity, and in particular M1 and M2 receptors which represent over 60 % of the brains cholinergic receptors, are vital in cognitive function [61]. The only antimuscarinic which was accorded a beneficial safety profile in the elderly is Fesoterodine, as this drug was studied specifically in the ageing population [62–64]. In the SOFIA trial 581 patients >65, of which 33 % were >75 years old and frequently on polypharmacy, completed a 3 month double-blind randomized trial of Fesoterodine versus placebo [62]. At 12 weeks, patients in the treatment arm demonstrated reduced urgency (−3.8 episodes), pollakiuria and nycturia (−0.55 episodes) (all *p* < 0.001) compared to placeebo. Fesoterodine determined a similar rate of adverse events compared to placebo (39.8 % vs 36.1 %), mostly mild xerostomia. Of note, no clinically relevant changes in cognitive function (evaluated through the mini-mental status examination) were observed throughout the study in both arms. This may be attributed to the high affinity of Fesoterodine for the M3 receptor and its inability to pass the blood–brain barrier [65]. In any case, great care is advised with anticholinergic drugs and a high level of suspiciousness in case of cognitive deterioration while receiving treatment.

### Intraprostatic agents

In addition to classic oral therapy, medical agents may be injected directly in the prostate [66]. This is a promising minimally invasive approach in patients who are unresponsive to medical treatment, who experience debilitating AE or who are poor candidates for surgery. The rationale for this therapy is the ability of some agents to determine prostatic involution and promote apoptosis, thus shrinking prostatic volume and ameliorating LUTS [66]. In addition, these agents may modulate prostatic afferent nerves, reducing nociception and improving BPH-related symptoms. However, it must be remembered that profound ameliorations were seen also in the sham arms of RCTs evaluating intraprostatic injections [67]: as such, the results of such trials must always be redimensioned and relativized to the sham-control arm, rather than considering the absolute results.

Ethanol has been explored as agent for intraprostatic administration, with favorable results. Investigators found a significant reduction of IPSS and an amelioration of Q_max_ and QoL [68, 69]. However results are seldom durable, and patients frequently require re-treatment, which has been reported necessary in over 40 % of patients [70]. Intraprostatic botulinum toxin injection is a very promising and is being throughout fully explored. This neurotoxin exists in seven different subtypes, and the most widely used has been Onabotulinum toxin A. Though yet unclear, it has been hypothesized that this may enhance prostatic apoptosis, down-regulate α-receptors and modulate afferent signaling in the prostate [71]. Investigators have reported positive and significant improvement of LUTS in men with BPH treated by Onabotulinum toxin injection [72]. Generally, doses between 100U and 300U have been used during most trials but Arnouk et al. reported similar functional and safety results after injection of 100U and 200U [73]. To date the largest trial testing botulinum in BPH was recently published by Marberger et al. in a phase II placebo-controlled trial, enrolling 380 men [74]. Patients were randomized to receive 100U, 200U or 300U Onabotulinum toxin A or 0.9 % saline, and were followed for 72 weeks. The investigators found a meaningful improvement of BPH parameters after botulinum injection, including IPSS (Δ IPSS: 5.6 to 6.6, according to dose), Q_max_(Δ = 2.0 to 2.4 ml/s, according to dose) and QoL. However, a pronounced placebo effect was observed, with patients in the control arm experiencing a similar symptom amelioration, yielding non-significant differences in outcomes across the treatment and control arm. Overall, intraprostatic injections of botulinum toxin are well-tolerated, with a low rate of AE mainly associated with the administration of the drug (2 % prostatitis) [74] and not the compound itself. Moreover, no sexual AEs are reported, with full conservation of sexual potency [75].

### Future perspectives in the medical treatment of BPH

Research in the field of BPH therapy is continuously progressing. As our molecular understanding of bladder, prostatic, urethral anatomy and pathophysiology advances, so do the experimental studies and clinical trials exploring new drugs in this domain. In particular there is growing interest in the role of inflammation, the vitamin D receptor signaling pathway and the activity of β_3_-receptors in BPH-mediated LUTS.

Inflammation has been associated with BPH pathogenesis and progression, with multiple cytokines and inflammatory cells responsible for the increased risk of BPH determined by prostatic inflammation [76]. The COX pathway leads to the production of free radicals and consequent oxidative stress: as such, a possible therapeutic effect of non-steroidal anti-inflammatory drugs has been hypothesized [77, 78]. Di Silverio et al. found that the combination of finasteride and a COX-2 inhibitor, rofecoxib 25 mg/die, caused a significant improvement in IPSS score (*p* = 0.0001) and of Q_max_ (*p* = 0.03) compared to finasteride alone [77]. Moreover flavocoxid, an inhibitor of COX and 5-lipoxygenase enzymes, reduced prostate weight, increased the expression of Bax and caspase-9 mRNA (pro-apoptotic) and decreased that of Bcl-2 (anti-apoptotic) in mice with induced BPH [78]. Although COX inhibitors could have a future role in the management of BPH, clinical evidence is still lacking and their application in BPH must be considered experimental.

The vitamin D receptor (VDR) signaling pathway could be associated with BPH and LUTS [79, 80]. Investigators have found that VDR agonists, notably elocalcitol, a synthetic derivative of vitamin D3 that regulates cell proliferation and apoptosis may inhibit the androgen-dependent and androgen-independentprostatic cell proliferation [81]. It can also reduce IL-8 secretion by inflammatory cells in the prostate by targeting the NF-kB pathway [80]. Elocalcitol modulates bladder contractility by inhibiting the calcium-sensitizing RhoA/ROCK with a potential interest in storage symptoms control [82]. In a phase II RCT, Colli et al. treated 57 men with prostate volumes ≥40 ml with elocalcitol for 12 weeks, finding a significant reduction of prostate growth compared to placebo (−2.90 vs +4.32, *p* < 0.0001) [79]. However until now in humans, elocalcitol was demonstrated with a very good safety profile but only exhibited limited efficacy on LUTS in patients with BPH and overactive bladder. Recent data in animals reported the interest of association of elicalcitol with tolterodine [83]. Clinical experimentation is continuing to evaluate its potential role in LUTS due to BPH and OAB management.

In the bladder the predominant form of β-adrenoceptor is the β3-receptor subtype. Its activation is associated with increased bladder capacity without change in micturition pressure, residual volume, or voiding contraction [84]. Mirabegron is a β3-receptor agonist that has been successfully tested in male and female patients suffering from OAB without BOO [85, 86] and is now being evaluated also in men with associated BOO. Nitti et al. in a randomized, double-blind, phase II study, treated 200 men affected by BOO with mirabegron 50, 100 mg or placebo. Mirabegron 50 mg was effective in reducing urgency and frequency, without impairing Q_max_ and with a non-significant increase in PVR urine [87]. Otsuki et al. studied the response to mirabegron 50 mg in two groups of patient, newly diagnosed OAB and BPH related OAB unresponsive to antimuscarinics [88]. They showed a significant improvement of OAB Symptom Score and IPSS –QOL index, voiding symptoms with no significant difference on post-void residual, supporting the use of Mirabegron in second line after failure of antimuscarinics. A recent randomized controlled trial tested the add-on of Mirabegron 50 mg to 0.2 mg tamsulosine compared to tamsulosine alone, with a significant benefit on urgency, daytime frequency and quality of life index after 2 months of therapy [89]. Although the increase in post-void residual urine volume was significantly greater in the add-on group, AUR was observed only in one man. The results of these trials suggest that Mirabegron may be effective in reducing storage LUTS and safe in patients affected by BOO.

Ion channel transient receptor potential subtype melastatin 8 (TRPM8) is an important factor in the mechanism of detection of bladder filling, whose activation can activate the initiation of micturition. Ito et al. described their activity in a rat model, finding [90] that administration of the TRPM8 antagonist RQ-00203078 significantly increased bladder capacity and voided volume. Moreover, the activation of TRPM8 is enhanced by cold temperatures, as found by Uvin et al. [91], demonstrating the known empirical finding that cold temperatures worsen urgency. Although these findings represent important steps in the understanding of the physiology of micturition, their clinical relevance to date remains limited: TRPM8 antagonists (PF-05105679) have been tested in phase 1 trials, however given the generalized expression of these receptors, significant side effects were recorded including hypothermia [92], thus limiting their possible clinical application.

PRX302 is a PSA-activated bacterial protoxin which has the ability to bind to cellular membranes, where it creates transmembrane channels with consequent lytic cell death. After intriguing results in animal models where intraprostatic injection of PRX302 caused extensive, organ-confined prostatic shrinkage [93], this molecule has been tested in humans with favorable preliminary results. In a phase II trial, 18 men received intraprostatic injections of PRX302 [94]. After one year there was an average change from baseline IPPS of −9.7 and of +2.8 ml/s inQ_max_. Moreover, 12/18 (67 %) patients showed a ≥20 % reduction inprostate volume at day 90. Of note, no patient experienced sexual AEs. Clearly, though these results appear very encouraging, the small sample size limits their interpretation and PRX302 is still considered experimental.

NX-1207 is another protein for intraprostatic injection currently under evaluation in preliminary studies. This molecule promotes focal apoptosis, with significant reductions of prostate volumes in animal models [95]. Human phase II studies found that intraprostatic injection of NX-1207 determined a reduction of AUA Symptom Score, maintained during 6 months follow-up, with no significant AE [95]. Two phase III trials are underway and their results are awaited to better analyze the true potential of this drug in BPH management.

## Conclusions

Today, BPH should not be considered a strictly prostatic disease, as it has been demonstrated that the entire lower urinary tract is involved in a complex pathophysiology. New medical treatments are available and the right drugs should be prescribed t the correct patients. Silodosin has similar efficacy compared to tamsulosin, with a lower risk of cardiovascular AE, making it a good choice for older patients requiring α1-antagonists. Tadalafil improves BPH symptoms in men with and without ED, and could be considered especially when ED and BPH coexist. Antimuscarinics are effective on residual storage symptoms after α1-antagonist therapy and appears to be safe even in men with moderate BOO, though these patients should be strictly monitored with regular PVR measurements. Intraprostatic injections of onabotulinum toxin A are a promising minimally invasive option for LUTS management, although their true efficacy is still object of evaluation. Finally, research in the field of BPH medical treatment is actively progressing, with new agents as elocalcitol and mirabegron being tested. Future basic research and prospective clinical trials must continue in order to increase our pharmacologic armamentarium for men suffering from LUTS, in order to reduce BPH progression, improve QoL and decrease AEs.

## Abbreviations

AE: Adverse effects
AUR: Acute urinary retention
BOO: Bladder outlet obstruction
BPH: Benign prostatic hyperplasia
cGMP: Cyclic guanosine monophosphate
ED: Erectile dysfonction
IPSS: International prostatic symptom score
LUTS: Lower urinary tract symtoms
NO: Nitric oxide
OAB: Overactive bladder
PDE5: Phosphodiesterase 5
QoL: Quality of Life
UI: Urinary infection
